# Fabrication of *Carum copticum* essential oil–loaded chitosan nanoparticles and evaluation its insecticidal activity for controlling *Rhyzopertha dominica* and *Tribolium confusum*

**DOI:** 10.3389/fpls.2023.1187616

**Published:** 2023-07-28

**Authors:** Masumeh Ziaee, Asiyeh Sheikhzadeh Takabi, Asgar Ebadollahi

**Affiliations:** ^1^ Department of Plant Protection, Faculty of Agriculture, Shahid Chamran University of Ahvaz, Ahvaz, Iran; ^2^ Department of Chemistry, Faculty of Science, Shahid Chamran University of Ahvaz, Ahvaz, Iran; ^3^ Department of Plant Sciences, Moghan College of Agriculture and Natural Resources, University of Mohaghegh Ardabili, Ardabil, Iran

**Keywords:** chitosan, essential oil, loading efficiency, nanoparticles, storage beetles

## Abstract

**Introduction:**

Plant essential oils (EOs) can be used as a feasible tool for insect pest control. Nanoparticle formulations of plant EOs can improve the efficiency and stability of EOs, as well as insecticidal potential.

**Methods:**

In this study, *Carum copticum* L. essential oil–loaded nanoparticles (OLNs) were prepared via an oil-in-water emulsion, followed by droplet solidiffication via ionic gelation using a cross-linker, sodium tripolyphosphate (TPP). The nanoparticles were characterized by ultraviolet and visible (UV–Vis) spectrophotometry, Fourier-transform infrared spectroscopy (FTIR), laser light scattering (LS), transmission electron microscopy (TEM), and scanning electron microscopy (SEM). Moreover, the insecticidal activity of *C. copticum* EO and OLNs was evaluated against *Rhyzopertha dominica* (F.) (Coleoptera: Bostrychidae) and *Tribolium confusum* Jacquelin du Val. (Coleoptera: Tenebrionidae). In addition, their effectiveness was assessed on the progeny production of tested insect species.

**Results and discussion:**

The loading efficiency ranged from 34.33 to 84.16% when the chitosan to EO weight ratio was 1:1.25 and 1:0.5, respectively. The loading efficiency decreased with increasing EO content in the nanoparticles. The OLN particles exhibited spherical shape. The particle size *was* in the range 120–223.6 nm and increased with the increase of EO to chitosan ratio. So that the largest mean particle size (223.6 nm) was reported in the 1:1.25 weight ratio of chitosan to the EO. The mortality percentage of *R. dominica* and *T. confusum* adults were 74 and 57% when exposed for 7 days to 2000 mg/kg of OLNs at the 1:1.25 weight ratio, while EO caused 62 and 44% mortality on both insect species, respectively. Therefore, OLNs can potentially improve the insecticidal activity of *C. copticum* EO and could be applied to facilitate control of stored-product insect pests.

## 1 Introduction

The lesser grain borer, *Rhyzopertha dominica* (F.) (Coleoptera: Bostrychidae) is a primary pest and generally infests stored wheat and other cereals but prefer wheat, corn, or rough and brown rice (Mason and McDonough, 2012). Both larvae and adults feed inside kernels, reducing them to hollow husks. Damaged kernels lose weight and their market value (Rees, 2007). The confused flour beetle, *Tribolium confusum* Jacquelin du Val. (Coleoptera: Tenebrionidae) is also one of the most destructive storage beetles of stored wheat in Iran and worldwide. Both adults and larvae infest cereals grains, grain products, and damage causes loss of weight and reduction in product volume. Moreover, the grains become contaminated with insects’ exuviae, faecal matter, and fragments (frass) (Hill, 2002). Therefore, the protection of stored products from insect pests’ infestation is the concern of the government, farmers, and those involved in this matter.

Maintaining pest prevention and control is an essential issue in reducing any type of damage (Kumar and Kalita, 2017). Various chemical pesticides such as malathion, bromophos, fenitrothion (Lemon, 1967), malathion, pirimiphos methyl (Shawir et al., 1988; Kljajić and Perić, 2007), cyfluthrin (Arthur, 1994; Arthur, 1998), thiamethoxam (Arthur et al., 2004), spinosad (Toews et al., 2003; Bonjour and Opit, 2010; Subramanyam et al., 2012), deltamethrin (Kljajić and Perić, 2007; Sehgal and Subramanyam, 2014; Ziaee and Babamir-Satehi, 2019), methoprene (Daglish and Wallbank, 2005; Athanassiou et al., 2011; Wijayaratne et al., 2012), and chlorfenapyr (Arthur, 2008; Arthur, 2009) have been used to control stored-product beetles. For decades, the use of chemical pesticides was one of the main components of the integrated management of storage insect and mite pests, providing long-term protection of stored products (Hamel et al., 2020). However, the adverse effects of pesticides on beneficial insects and non-target organisms, the risk of synthetic pesticides residues in products, the occurrence of resistance in insect pests, and the risk of the environmental pollution have caused to increase in the tendency to use safer compounds for insect pest control (Damalas and Eleftherohorinos, 2011; Barzman et al., 2015). Therefore, investigating the appropriate, safe, and economical methods, such as botanical insecticides, can be effective in the management of insects (Hikal et al., 2017; Ahmed et al., 2021). Botanical insecticides, namely, pyrethrum, neem, and insecticides based on plant essential oils (EOs) and plant extracts are commercially produced and entered the marketplace (Isman, 2006; Rharrabe et al., 2008; Adarkwah et al., 2010; Dively et al., 2020; Moldovan et al., 2020). Among botanical insecticides, plant EOs have advantages such as low toxicity on mammals, fast degradability, and local availability (Isman, 2004). Some of the plant essential oils and their compounds have insecticidal, repellent, and antifeedant properties on insects. Therefore, they can be used as an alternative to chemical insecticides to protect agricultural crops (Isman, 2006; Said-Al Ahl et al., 2017). Encapsulation can protect active agents from severe conditions, for example, light, oxygen, and heat (Yinbin et al., 2018). Moreover, this process causes a slow and controlled release of the loaded compound to prolong its effectiveness (Yoksan et al., 2010). Therefore, to overcome the issue of plant EOs low stability, these compounds be applied in different formulations such as nanoparticles, nano- and microcapsules, nano- and microemulsions, and so forth; and be used in integrated pest management programs (Ebadollahi et al., 2021; Devrnja et al., 2022).

Iran is rich in medicinal and aromatic plants, and more plants contain various chemical compounds and biological activity (Hassanpouraghdam et al., 2022). Ajwain, *Carum copticum* L. (Apiaceae), is a traditional medicinal plant. Ajwain has small white flowers and brown fruit, and there are five thin longitudinal lines in light yellow color on the surface of the fruit. The seeds of *C. copticum* are rich in fiber, minerals, vitamins, and antioxidants and have many medicinal uses (Boskabady et al., 2014). In our previous study, the toxicity of *C. copticum* EO-loaded nanogels was reported against *Sitophilus granarius* (L.) (Coleoptera: Curculionidae) and *T. confusum* adults. Moreover, the EO persisted for up to 20 days when loaded in nanogels (Ziaee et al., 2014). Various techniques can be used to encapsulate EOs, which can cause changes in the oil efficiency, and potential activity against insect pests (Maes et al., 2019). Therefore, in this study *C. copticum* essential oil–loaded nanoparticles (OLNs) was synthesized by two-step procedure, that is, droplet constitution and droplet solidification via ionic gelation; then physicochemical characterizations of the OLNs was evaluated considering the following parameters: ultraviolet and visible (UV–Vis) spectrophotometry, Fourier-transform infrared spectroscopy (FTIR), laser light scattering (LS), transmission electron microscopy (TEM), and scanning electron microscopy (SEM). Moreover, the insecticidal activity of the EO and OLNs and effects on the progeny production of *R. dominica* and *T. confusum* were evaluated.

## 2 Materials and methods

### 2.1 Insect rearing

The colony of lesser grain borer, *R. dominica*, and the confused flour beetle, *T. confusum* were obtained from cultures kept in the toxicology laboratory at the Shahid Chamran University of Ahvaz, Ahvaz, Iran, for at least 3 years. *Rhyzopertha dominica* was reared on whole wheat (variety Chamran), and *T. confusum* was reared on a diet containing a mixture of wheat flour and brewer yeast (10:1 w:w). The rearing conditions were 27 ± 1°C and 65 ± 5% relative humidity (RH) in continuous darkness. Unsexed adults (7–14 days old) were used for the bioassays.

### 2.2 *Carum copticum* EO extraction

*Carum copticum* seeds were purchased from a local market in Mashhad, Iran. The seeds were ground and hydrodistilled for 4h using a clevenger-type apparatus at 100°C to extract the EO. Anhydrous sodium sulphate was used to remove water, and the obtained oil was kept in a refrigerator at 4°C. The density of *C. copticum* EO was measured as 0.947 g/L.

### 2.3 Chitosan-based nanoparticles preparation loaded with *Carum copticum* EO

The nanoparticles were synthesized with the technique of Keawchaoon and Yoksan (2011) and Ahmadi et al. (2018) with some modifications. Chitosan (Mw = 340 g/mol) was purchased from Sigma-Aldrich Chemicals Co. (Saint Louis, MO, USA). Tween 80 and sodium tripolyphosphate (TPP) were purchased from Merck, Germany. Chitosan (1.2% w/v) was dissolved in 40 mL of acetic acid solution (1% v/v) under a magnetic stirrer for 20 min. The emulsifier Tween 80 (HLB: 15.0, 0.306 g) was added to the chitosan solution and stirred for 1h until a homogeneous solution was obtained. The EO was added in different ratios, namely, 0, 0.24, 0.48, and 0.60 g, to provide the weight ratios of chitosan to oil of 1:0, 1:0.50, 1:1, and 1:1.25, respectively. The oil was added to the solution and stirred for 20 min at a speed of 500 rpm. Subsequently, the TPP solution was prepared separately by dissolving TPP (0.5% w/v), and the TPP solution was added drop wisely to the EO solution loaded with chitosan Tween 80 and stirred at a speed of 500 rpm for 30 min. The solution was centrifuged at 10,000 rpm, 5°C for 10 min, and washed with distilled water three times to separate the unloaded oil (*C. copticum* oil). The prepared suspension was freeze-dried using freeze dryer (ALPHA 1-2 LD plus, Christ Co. Germany) at −35°C for 72h.

### 2.4 Characterization of *Carum copticum* EO-loaded nanoparticles

#### 2.4.1 Fourier-transform infrared spectroscopy

The chemical structure of all used materials, including Tween 80, TPP, chitosan, *C. copticum* EO, and OLNs was characterized by the FTIR technique. FTIR spectra were recorded as KBr discs using a PerkinElmer FTIR spectrometer (USA) at a resolution of 4 cm^−1^ from 4000 to 400 cm^−1^.

#### 2.4.2 EO-loading efficiency

The unloaded nanoparticles were considered as a blank for basic corrections. The colorimetric assay at 273 nm was carried out for absorbency, and the spectrum was collected at 200–400 nm (Ziaee et al., 2014). To separate the unloaded oil (supernatant) from prepared nanoparticles, oil-loaded nanoparticles were centrifuged at 2684*g*, 5°C for 10 min. The absorbency of the solution was determined at 273 nm by UV–VIS spectrophotometer (UNICO Model 2100 series, Dayton, NJ, USA), and the result was compared with that of the standard curve. The process was replicated three times. The loading efficiency (LE) of EO was calculated using Liu et al. (2005) equation:


$$LE=\frac{\text{mass of oil added into the solution}}{\text{mass of oil in supernatant after centrifugation }}\times 100$$

#### 2.4.3 Particle size and morphology of *Carum copticum* EO-loaded nanoparticles

An LS instrument (Scatterscop Qudix, Seoul, South Korea) was used to determine the median particle size and size distribution of nanoparticles. Dynamic light scattering was performed at a 90° and at temperature of 25°C. All samples were analyzed in triplicate, and their average was reported. TEM was performed using the Transmission electron microscope (Zeiss LEO 906 E, Freiburgim Breisgau, Germany) at an accelerating voltage of 80 kV. For this purpose, the samples were prepared by depositing a drop of nanoparticles containing phosphotungstic acid (2%) onto copper grids, and the extra liquid was removed by a filter paper. Then, the grids were allowed to air dry at room temperature. The structure of nanoparticles was investigated using SEM (Zeiss LEO 1455 VP, Freiburg im Breisgau, Germany) at 30k V acceleration voltage.

### 2.5 Insecticidal activity of chitosan-based nanoparticles loaded with *Carum copticum* EO

The insecticidal activity of EO and OLNs was assessed to protect wheat grains (Chamran variety, 11% moisture content) against *R. dominica* and *T. confusum* adults. Wheat grains (100 g) were poured into 250-mL glass jars and treated with 1000 and 2000 mg/kg of EO or OLNs for 1:0.50, 1:1, and 1:1.25 ratios of chitosan to oil. The concentration of 1000 mg/kg was equal to 195.7, 327.4, and 378.3 mg/kg EO and 2000 mg/kg was equal to 391.4, 654.8, and 756.6 mg/kg EO for 1:0.50, 1:1, and 1:1.25 ratios of chitosan to oil, respectively. The untreated wheat grains and treated with unloaded nanoparticles were considered as the negative and positive control groups, respectively. Caps were screwed and jars were shaken for 3 min to distribute the nanoparticles in the entire wheat grains. Subsequently, 20 adults were placed in each jar separately. The jars were kept in darkness at 27°C and 60% RH. The treated and untreated wheat grains were replicated five times and arranged in a completely randomized design. Adult mortality was recorded 2 and 7 days after exposure, whereas progeny was recorded after 65 days.

### 2.6 Statistical analysis

The mortality and progeny data were checked for normality using Shapiro–Wilk’s test at *P* = 0.05. No mortality was reported in the negative control group (untreated wheat grains) of both species, so there was no need to correct mortality counts. Mortality data, for each exposure time, and progeny data were subjected to one-way ANOVA. Means were separated by Tukey–Kramer (HSD) test. In addition, at each concentration level, independent sample t-test was used to determine whether there was a significant difference between EO and OLNS (Sokal and Rohlf, 1995). All statistical analysis were performed using SPSS software version 16.0 at *P* = 0.05 (IBMCorp, 2007).

## 3 Results

### 3.1 Chitosan-based nanoparticles preparation loaded with *Carum copticum* EO

**Figure 1:**
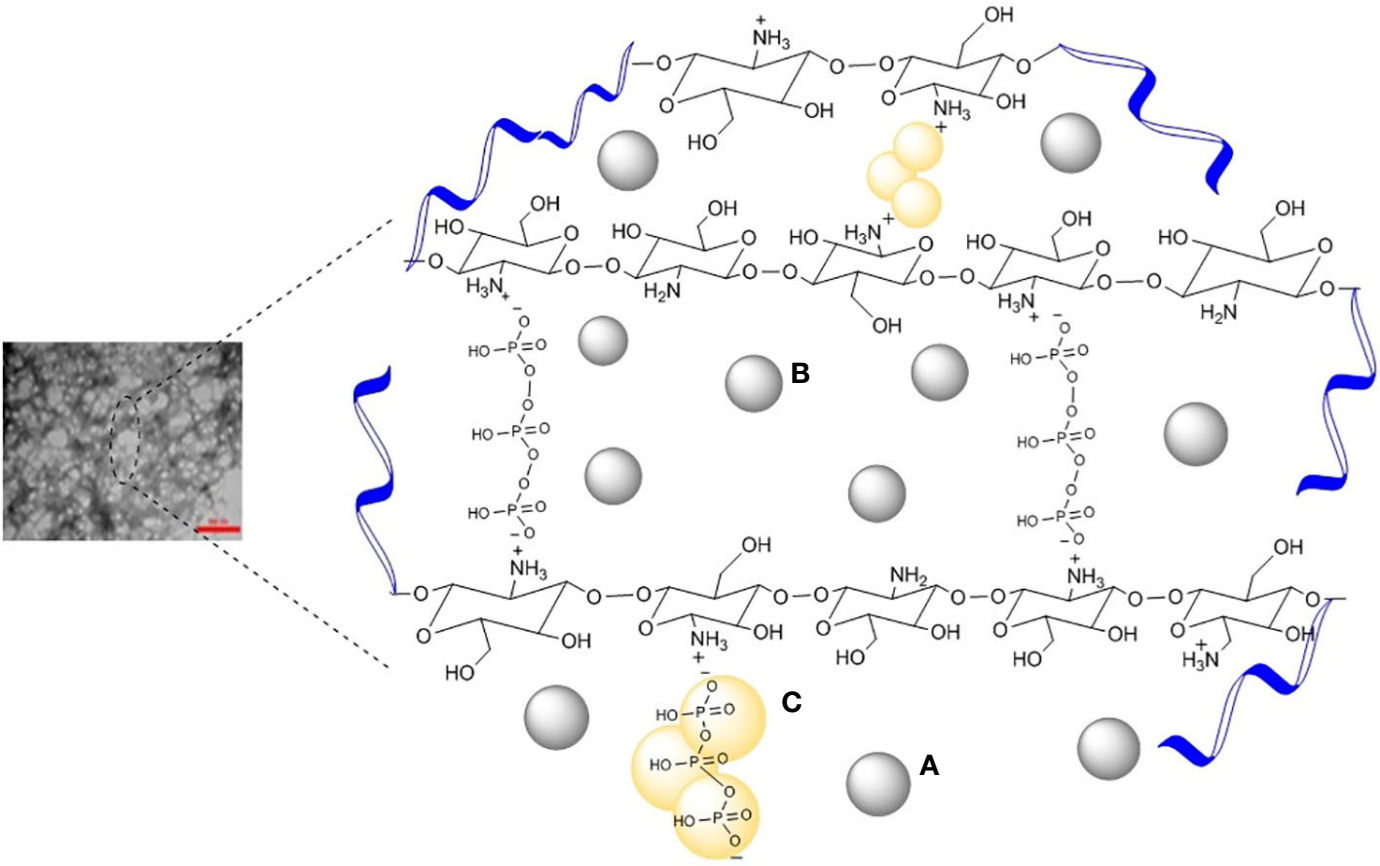
Schematic illustration of **(A)** oil in chitosan droplets, **(B)** oil-loaded chitosan particles, and **(C)** chemical structure of chitosan ionically cross-linked with TPP.

The *C. copticum* EO loaded-chitosan/TPP nanoparticles were synthesized by a two-step procedure, that is, droplet constitution and droplet stability. The constitution of the EO droplets in chitosan solution was obtained by an oil-in-water emulsion. The TPP is present in the bulk of the nanoparticles that are bridged between the positive charges of the chitosan chains. The negatively charged *C. copticum* EO, on the other hand, linked chitosan on the surface of the nanoparticles. Each droplet was placed by ionic cross-linking of protonated amino groups (NH_3_^+^) along chitosan molecules circumambient the *C. copticum* EO droplet and polyphosphate groups (P_3_O_10_^5−^) of TPP molecules (**Figure 1**).

### 3.2 Fourier-transform infrared spectroscopy

**Figure 2:**
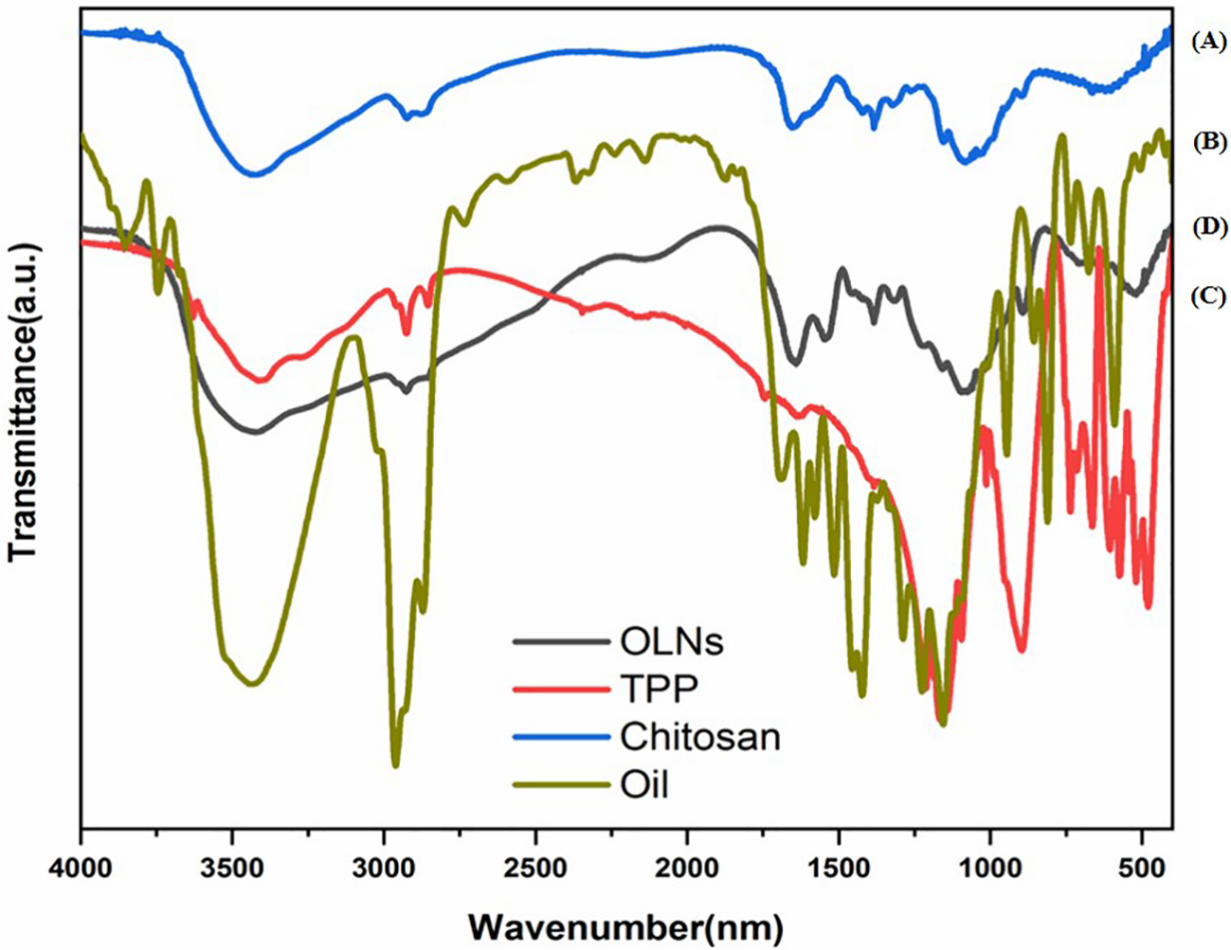
FTIR spectra of **(A)** chitosan particles, **(B)** *Carum copticum* EO, **(C)** TPP, and **(D)** OLNs (oil-loaded chitosan nanoparticles with chitosan to EO weight ratio of 1:0.5).

**Figure 2** shows FTIR spectra of chitosan particles, TPP, *C. copticum* oil, and oil-loaded chitosan nanoparticles. In general, chitosan particles show characteristic peaks at 3435 cm^−1^ (OH and NH_2_ stretching), 2923 cm^−1^ (CH stretching), 1655 cm^−1^ (NH bending), 1088 cm^−1^ (C-O-C, C-N stretching), and 591 cm^−1^ (pyranoside ring stretching vibration), and new peaks appeared around 1316–1076 cm^−1^ (P─O and P═O) (**Figure 2A**). The *C. copticum* EO shows that characteristic peaks at 3450 cm^−1^ refer to the stretching vibrations of the OH group of the EO. The peak at 3020 cm^−1^ is related to the stretching vibrations of the CH group of aromatic compounds in the EO. Characteristic peaks were at 2925 cm^−1^ (CH stretching), 1589 cm^−1^ (NH bending), and 1384 cm^−1^ (**Figure 2B**). TPP spectra indicates 1211 cm^−1^ (P═O stretching), 1127 cm^−1^ (symmetric and antisymmetric stretching vibrations in PO_2_ group), 1093 cm^−1^ (symmetric and antisymmetric stretching vibrations in PO_3_ group), and 800 cm^−1^ (antisymmetric stretching of the P─O─P bridge) (**Figure 2C**). Moreover, in comparison with the FTIR spectra of chitosan particles, the addition of EO resulted in a marked increase in the intensity of the CH stretching peak at 2867–2925 cm^−1^ (**Figure 2D**).

### 3.3 EO-loading efficiency

About 84% of the EO was loaded into nanoparticles when the weight ratio of chitosan to oil was 1:0.5; the percentage of loaded *C. copticum* oil was in the range of 34.33–84.16%. The mean particle size of OLNs ranged from 120.0, when no oil was loaded in the nanoparticles, to 223.6 for the weight ratio of 1:1.25 (chitosan to oil) (**Table 1**).

**Table 1:** Loading efficiency (LE) % ( ± SE) and mean particle size of *Carum copticum* OLNs.

Chitosan to oil weight ratios	LE (%)	Mean particle size (nm)
1:0 (control)	–	120.0 ± 6.93b
1:0.5	84.16 ± 1.64a	125.3 ± 9.02b
1:1	71.16 ± 0.72b	138.3 ± 12.57b
1:1.25	34.33 ± 0.44c	223.6 ± 11.40a
df _treatment, error_	2, 6	3, 8
*F*, *P*	586.691,< 0.001	22.531,< 0.001

Means followed by the same lowercase letter within each column are not significantly different using Tukey-Kramer (HSD) test at 0.05.

### 3.4 Particle size and morphology of *Carum copticum* EO-loaded nanoparticles

The average size of the oil-loaded nanoparticles ranged from 125.3 to 223.6 nm using the LS technique. The size of the particles was increased with increasing oil content from 0.24 to 0.6 g in chitosan particles (**Table 1**).

**Figure 3:**
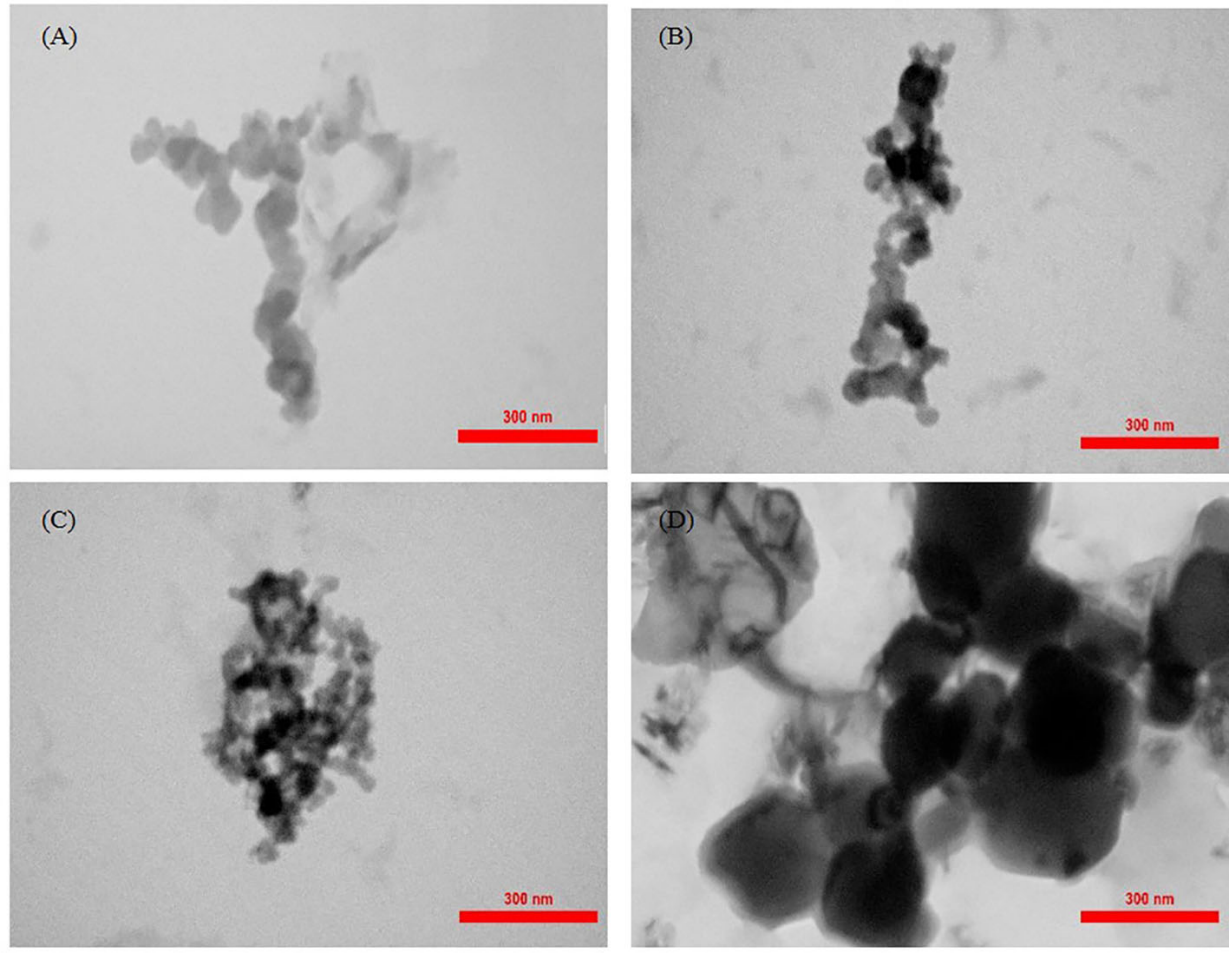
TEM micrographs at 80 kV of *Carum copticum* oil–loaded nanoparticles using an initial weight ratio of chitosan to oil of **(A)** 1:0, **(B)** 1:0.5, **(C)** 1:1, and **(D)** 1:1.25.

**Figure 4:**
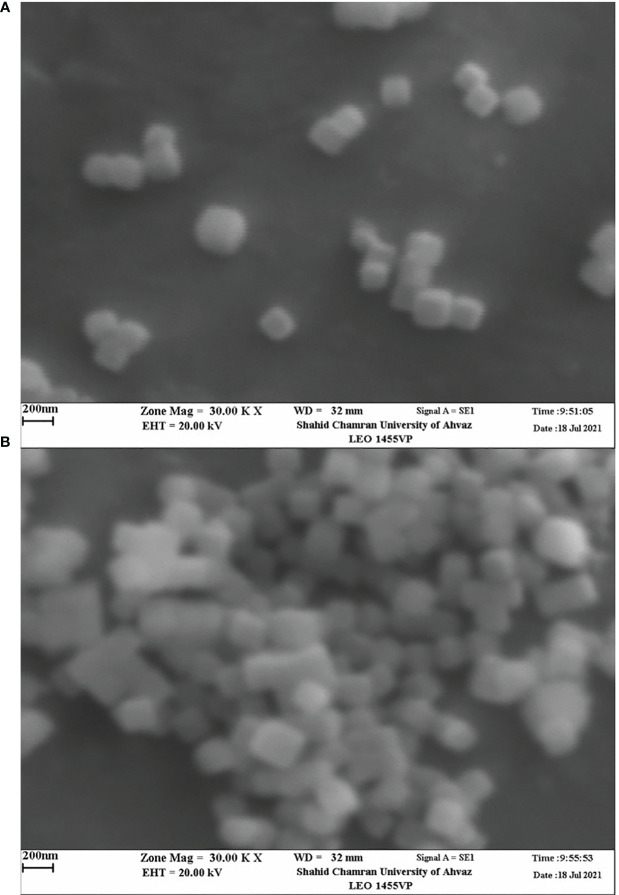
SEM micrographs of *Carum copticum* oil–loaded nanoparticles using an initial weight ratio of chitosan to oil of **(A)** 1:0.5, and **(B)** 1:1.

The morphology of nanoparticles was observed by TEM and SEM analysis (**Figures 3**, **4**). The unloaded and OLNs had a spherical shape. The unloaded nanoparticles were lighter in color, which indicated the absence of EO inside these particles (**Figure 3A**). Moreover, the size of nanoparticles increased by increasing the EO content. In all samples, individual particles form bonds with each other resulting in aggregates (**Figures 3B-D**). SEM images indicated that chitosan particles have a nearly smooth and spherical appearance (**Figure 4**).

### 3.5 Insecticidal activity of chitosan-based nanoparticles loaded with *Carum copticum* EO

The mortality percentage of *R. dominica* adults exposed to EO and OLNs in concentrations of 1000 and 2000 mg/kg is presented in **Table 2**. At a concentration of 1000 mg/kg, there was significant differences among different treatments of EO (*F_3, 16 =_* 165.744; *P<* 0.001) and OLNs (*F_3, 16 =_* 138.458; *P<* 0.001), and 1:1.25 weight ratio was the most effective in controlling *R. dominica* adults, 2 days after exposure. The higher susceptibility was recorded when *R. dominica* adults were exposed to the EO than OLNs, 2 days after exposure. Although, the adults’ mortality increased overtime 7 days after treatment and there was significant differences among different treatments of EO (*F_3, 16 =_* 119.538; *P<* 0.001) and OLNs (*F_3, 16 =_* 82.5877; *P<* 0.001). At this time interval, the mortality percentage of *R. dominica* adults was significantly higher in wheat treated with OLNs compared with the control and EO. In addition, at the concentration of 2000 mg/kg, there were significant differences among different treatments of EO (*F_3, 16 =_* 475.933; *P<* 0.001) and OLNs (*F_3, 16 =_* 187.143; *P<* 0.001) in controlling *R. dominica* adults, 2 days after exposure. The toxicity of OLNs was significantly higher than EO against *R. dominica* adults leading to 54, 67, and 74% mortality after 7 days of exposure to chitosan nanoparticles at 1:0.5, 1:1, and 1:1.25 chitosan to oil weight ratios, respectively. While at this concentration, the EO caused 43, 56, and 62% mortality in the tested species (**Table 2**).

**Table 2:** Mean mortality % ( ± SE) of *Rhyzopertha dominica* exposed to wheat treated with *Carum copticum* EO and OLNS.

Time (day)	Chitosan to EO weight ratios	1000 mg/kg		*t_8_*	2000 mg/kg		*t_8_*
		EO	OLNS		EO	OLNS	
2	1:0 (control)	0.00 ± 0.00d	0.00 ± 0.00d	–	0.00 ± 0.00d	0.00 ± 0.00d	–
	1:0.5	20.0 ± 1.58c	18.0 ± 1.22c	1.00	39.0 ± 1.0c	36.0 ± 1.87c	1.414
	1:1	30.0 ± 1.58b*	24.0 ± 1.87b	2.449	47.0 ± 1.22b	45.0 ± 1.58b	1.00
	1:1.25	38.0 ± 1.22a*	33.0 ± 1.22a	2.887	55.0 ± 1.58a	57.0 ± 1.22a	1.00
	*F_3, 16_*	165.744	138.458		475.933	187.143	
	*P*	< 0.001	< 0.001		< 0.001	< 0.001	
*7*	1:0	5.0 ± 1.58d	5.0 ± 2.23c	0.001	5.0 ± 1.58c	5.0 ± 2.23c	0.001
	1:0.5	24.0 ± 1.87c	36.0 ± 2.50b*	3.893	43.0 ± 1.22b	54.0 ± 1.87b*	4.919
	1:1	34.0 ± 1.0b	47.0 ± 1.22a*	8.222	56.0 ± 1.00a	67.0 ± 2.54a*	4.017
	1:1.25	44.0 ± 1.0a	51.0 ± 2.91a*	2.271	62.0 ± 3.0a	74.0 ± 1.87a*	3.394
	*F_3, 16_*	119.538	82.5877		321.600	209.441	
	*P*	< 0.001	< 0.001		< 0.001	< 0.001	

For each exposure time, means followed by the same lower case letter within each column are not significantly different using Tukey–Kramer (HSD) test at 0.05. For each ratio, differences between EO and OLNS denoted with an asterisk indicate a significant difference using t-student test at 0.05. Where no letters exist, no signiﬁcant differences were noted.

The mortality percentage of *T. confusum* adults exposed to EO and OLNs is presented in **Table 3**. The EO caused moderate mortality at a low concentration of 1000 mg/kg against *T. confusum* adults, and there was significant differences among different treatments of EO (*F_3, 16 =_* 217.524; *P<* 0.001) and OLNs (*F_3, 16 =_* 84.923; *P<* 0.001), 2 days after exposure. The mortality did not exceed 32% after 2 days of exposure to EO and reached 38% after 7 days of adults’ exposure. At 7-day exposure time, there were significant differences among different treatments of EO (*F_3, 16 =_* 97.125; *P<* 0.001) and OLNs (*F_3, 16 =_* 183.792; *P<* 0.001). In the case of OLNs, the mortality was 27% at the highest oil content after 2 days of exposure, while the insecticidal activity of nanoparticles increased over time, and 47% mortality was reported after 7 days of exposure. At 2000 mg/kg of EO, adult mortality was 38% after 2 days of exposure (*F_3, 16 =_* 109.467; *P<* 0.001), while it reached 44% after 7 days (*F_3, 16 =_* 196.121; *P<* 0.001). In addition, there were significant differences among different treatments of EO (*F_3, 16 =_* 73.750; *P<* 0.001) and OLNs (*F_3, 16 =_* 186.051; *P<* 0.001) in controlling *T. confusum* adults, 7 days after exposure. The highest *T. confusum* mortality (57%) was reported when adults were exposed to wheat grains treated with 2000 mg/kg OLNs at a 1:1.25 chitosan to oil weight ratio after 7 days of exposure (**Table 3**).

**Table 3:** Mean mortality % ( ± SE) of *Tribolium confusum* exposed to wheat treated with *Carum copticum* EO and OLNS.

Time (day)	Chitosan to EO weight ratios	1000 mg/kg		*t_8_*	2000 mg/kg		*t_8_*
		EO	OLNS		EO	OLNS	
2	1:0(control)	0.00 ± 0.00d	0.00 ± 0.00d	–	0.00 ± 0.00c	0.00 ± 0.00d	–
	1:0.50	14.0 ± 1.0c	12.0 ± 1.22c	1.265	25.0 ± 2.23b	25.0 ± 1.58c	0.001
	1:1	24.0 ± 1.0b	21.0 ± 1.87b	1.414	31.0 ± 1.87b	32.0 ± 1.22b	0.447
	1:1.25	32.0 ± 1.22a*	27.0 ± 1.22a	2.880	38.0 ± 1.22a	37.0 ± 1.22a	0.577
	*F_3, 16_*	217.524	84.923		109.467	196.121	
	*P*	< 0.001	< 0.001		< 0.001	< 0.001	
*7*	1:0	5.0 ± 1.58d	3.0 ± 2.00d	0.784	5.0 ± 1.58c	3.0 ± 2.00c	0.784
	1:0.50	20.0 ± 1.58c	32.0 ± 1.22c*	6.00	31.0 ± 2.44b	44.0 ± 1.00b*	4.914
	1:1	28.0 ± 1.22b	39.0 ± 1.00b*	6.957	38.0 ± 1.22ab	52.0 ± 2.54a*	4.950
	1:1.25	38.0 ± 1.22a	47.0 ± 1.22a*	5.196	44.0 ± 2.44a	57.0 ± 1.22a*	4.747
	*F_3, 16_*	97.125	183.792		73.750	186.051	
	*P*	< 0.001	< 0.001		< 0.001	< 0.001	

For each exposure time, means followed by the same lower case letter within each column are not significantly different using Tukey–Kramer (HSD) test at 0.05. For each ratio, differences between oil and OLNS denoted with an asterisk indicate a significant difference using t-student test at 0.05. Where no letters exist, no significant differences were noted.

In both tested species, the number of progeny in the treatments showed a significant decrease compared with the control. In addition, the number of progeny decreased significantly with increasing the ratio of EO to chitosan and concentration level. For *R. dominica*, there were significant differences among different treatments of EO at the concentration of 1000 mg/kg (*F_3, 16 =_* 101.014; *P<* 0.001) and 2000 mg/kg (*F_3, 16 =_* 165.083; *P<* 0.001). In most cases, OLNs significantly reduced the production of progeny than the EO in both storage beetles. The significant differences in progeny number were reported when *R. dominica* adults were exposed to 1000 mg/kg (*F_3, 16 =_* 143.467; *P<* 0.001) and 2000 mg/kg (*F_3, 16 =_* 191.440; *P<* 0.001) of OLNs. For *T. confusum*, significant differences were noted in the number of progeny found on wheat treated with OLNs at concentration level of 1000 mg/kg (*F_3, 16 =_* 300.848; *P<* 0.001) and 2000 mg/kg (*F_3, 16 =_* 409.529; *P<* 0.001) (**Table 4**).

**Table 4:** Mean progeny number ( ± SE) of *Rhyzopertha dominica* and *Tribolium confusum* exposed to wheat treated with *Carum copticum* EO and OLNS.

Insect species	Chitosan to EO weight ratios	1000 mg/kg		*t_8_*	2000 mg/kg		*t_8_*
		EO	OLNS		EO	OLNS	
*R. dominica*	1:0 (control)	15.8 ± 1.01a	20.4 ± 1.28a^*^	2.799	15.8 ± 1.01a	20.4 ± 1.28a^*^	2.799
	1:0.50	9.20 ± 0.49b^*^	4.80 ± 0.80b	4.690	7.20 ± 0.37b^*^	3.20 ± 0.58b	5.774
	1:1	2.40 ± 0.67c	2.0 ± 0.32bc	0.535	1.0 ± 0.32c*	0.0 ± 0.0c	3.162
	1:1.25	1.0 ± 0.32c^*^	0.0 ± 0.0c	3.162	0.0 ± 0.0c	0.0 ± 0.0c	–
	*F_3, 16_*	101.014	143.467		165.083	191.440	
	*P*	< 0.001	< 0.001		< 0.001	< 0.001	
*T. confusum*	1:0 (control)	9.60 ± 0.40a	11.80 ± 0.583a^*^	3.11	9.60 ± 0.40a	11.80 ± 0.583a^*^	3.11
	1:0.50	5.40 ± 0.40b^*^	1.00 ± 0.32b	8.629	1.40 ± 0.25b^*^	0.0 ± 0.0b	5.715
	1:1	3.00 ± 0.32c^*^	0.0 ± 0.0b	9.487	0.40 ± 0.24bc	0.0 ± 0.0b	1.633
	1:1.25	0.80 ± 0.37d	0.0 ± 0.0b	2.138	0.0 ± 0.0c	0.0 ± 0.0b	–
	*F_3, 16_*	101.429	300.848		294.238	409.529	
	*P*	< 0.001	< 0.001		< 0.001	< 0.001	

For each insect species, means followed by the same lower case letter within each column are not significantly different using Tukey–Kramer (HSD) test at 0.05. For each ratio, differences between EO and OLNS denoted with an asterisk indicate a significant difference using t-student test at 0.05. Where no letters exist, no signiﬁcant differences were noted.

## 4 Discussion

The loading percentage decreased with the increase in the weight ratio of EO to chitosan. It is in accordance with the findings of Keawchaoon and Yoksan (2011), who reported increases in LE with increasing initial carvacrol content in oil-loaded chitosan/TPP nanoparticles. Decreases in the LE of oregano EO in chitosan/TPP nanoparticles with increasing the weight ratio of EO to chitosan was reported by Hosseini et al. (2013), in which the highest efficiency percentage was detected at the 1:0.1 weight ratio of chitosan to EO. The decrease in LE of the nanoparticles can be due to the saturation of the EO loading into chitosan nanoparticles (Hosseini et al., 2013), as well as limitation of EO loading in chitosan nanoparticles (Esmaeili and Asgari, 2015).

In our study, chitosan particles show peaks at 3435 cm^−1^ (OH and NH_2_ stretching), 2923 cm^−1^ (CH stretching), 1655 cm^−1^ (NH bending), 1088 cm^−1^ (C─O─C, C─N stretching), and 591 cm^−1^ (pyranoside ring stretching vibration). Keawchaoon and Yoksan (2011) reported the peaks at 3500–3250 (OH), 2927 (CH stretching), 1634 (amide I), 1539 (amide II), 1155 (P O) [39–41], 1072 (COC), and 890 cm−1 (pyranose ring) in chitosan nanoparticles. In addition, chitosan particles shows new peaks at 1316–1076 cm^−1^ (P─O and P═O), this peak implying the complex formation via electrostatic interaction between phosphoric groups of TPP and NH_3_^+^ ions within the nanoparticles (Yoksan et al., 2010). The *C. copticum* oil-loaded chitosan nanoparticles showed similar FTIR spectra to that of chitosan particles. It was revealed that the *C. copticum* EO has been loaded into the chitosan nanoparticles without any chemical reaction. Therefore, the structure and function of the EO have not changed in the process of synthesizing nanoparticles; as a result, its insecticidal activity. The CH stretching peak at 2867–2925 cm^−1^, indicating an increase in the content of ester groups, which might come from EO molecules. The use of CH stretching peak as a probe band to determine the loaded EO content was in accordance with Keawchaoon and Yoksan (2011) research, who reported that the increase in the intensity of CH stretching at 2870–2959 cm^−1^ in oil-loaded chitosan/TPP nanoparticles indicating the presence of carvacrol oil in the chitosan nanoparticles.

Chitosan nanoparticles loaded with oregano EO exhibited a regular distribution and spherical shape with a size range of 40–80 nm. The size of unloaded chitosan nanoparticles was smaller than the oil-loaded ones, which may be attributed to the presence of oil in the particles (Hosseini et al., 2013). The size of the chitosan particles increased with the amount of EO in nanoparticles (Keawchaoon and Yoksan, 2011). The mean particle size of oregano EO-loaded chitosan particles increased as an increase in initial OEO content (Hosseini et al., 2013). Moreover, our findings showed that the larger particles might be according to the agglomeration of the particles. TEM images also confirmed the aggregation of particles, which increased the particle size. It was pointed out that the larger diameter of particles might result from the swelling chitosan layer surrounding the individual chitosan particles (Yoksan et al., 2010; Keawchaoon and Yoksan, 2011). Ahmadi et al. (2018) prepared *Achillea millefolium* (L.) oil-loaded chitosan nanocapsules with spherical shapes and a compact structure. In our study, SEM images of the chitosan nanoparticles loaded with *C. copticum* EO demonstrated the regular distribution of the particles and spherical shape with smooth surfaces. Spherical with smooth surface particles were obtained when chitosan nanoparticles were functionalized with β-cyclodextrin containing carvacrol and linalool. The smooth surface indicates the absence of pores on the surface of the nanoparticles, which can improve protection against degradation and volatilization processes under environmental conditions (Campos et al., 2018).

The effects of the *C. copticum* oil-loaded nanoparticles were evaluated considering the mortality percentage of *R. dominica* and *T. confusum* adults in wheat grains treated with the formulations. All the treatments resulted in significantly higher mortality than the negative (untreated wheat) and positive (wheat treated with unloaded chitosan nanoparticles) control groups. EO of *C. copticum* has been reported as an effective botanical insecticide against stored-product insect pests (Sahaf et al., 2007; Upadhyay et al., 2007; Sahaf and Moharramipour, 2008; Shojaaddini et al., 2008; Habashi et al., 2011; Ziaee et al., 2014). Sahaf et al. (2007) documented high fumigant toxicity of *C. copticum* EO against *Sitophilus oryzae* (L.) (Coleoptera: Curculionidae) and *Tribolium castaneum* (Herbst) (Coleoptera: Tenebrionidae). They stated that *T. castaneum* was more tolerant than *S. oryzae*. The EO of *C. copticum* was noted to be highly effective against different life stages of *Plodia interpunctella* (Hubner) (Lepidoptera: Pyralidae) (Shojaaddini et al., 2008). Our results show that *C. copticum* EO has insecticidal potential against *R. dominica* and *T. confusum* adults, but the activity decreased with time. Developing the EO in chitosan nanoparticles prepared an effective alternative control agent for managing storage beetles. Our previous reports highlighted that myristic acid–chitosan nanogels loaded with *C. copticum* EO were highly toxic compared with the EO on *S. granarius* and *T. confusum* (Ziaee et al., 2014).

## 5 Conclusion

Chitosan nanoparticles provide an ideal delivery system for EO release. The present work successfully developed *C. copticum* EO-loaded chitosan particles, as confirmed by an absorption band at 273 nm (UV–VIS spectrophotometer). Moreover, FTIR spectra of the loaded nanoparticles indicated that the characteristic absorption peaks related to the presence of EO in the nanoparticles. The LE of the nanoparticles ranged from 34.33 to 84.16%. The particles were spherical with an average size of 125-223 nm, indicating that the size increased with an increasing amount of EO. The toxicity of OLNs against both tested storage beetles increased with increasing time at a 7-day time interval as a grain protectant. According to our results, the prepared formulation with the weight ratio of chitosan to the EO of 1:1 and a TPP concentration of 0.5% (w/v) was the optimal formulation. The results open windows for studies on the practical utilization of OLNs for managing stored-product insect pests. The fate of tested formulations and organoleptic properties of treated food materials should be considered before their application. Moreover, further research is necessary to assess the cytotoxic activity and bioavailability of OLNs. To commercialize the formulation containing plant EO as an active ingredient, the cost and economic feasibility of the formulation should be taken into consideration. Plants should be cultivated on a field, near the production facility to achieve cost-effective and large-scale production systems. One of the other challenges of commercializing this formulation is prolonging the residual effect of the active ingredient, which should be carefully studied. If all the limitations and challenges ahead for the commercialization of OLNs are overcome, we can have promising prospects for bio-insecticide production.

## Data availability statement

The original contributions presented in the study are included in the article/supplementary material. Further inquiries can be directed to the corresponding author.

## Ethics statement

Ethical review and approval was not required for the study on animals in accordance with the local legislation and institutional requirements.

## Author contributions

Conceptualization: MZ and AS, methodology: MZ and AS, investigation: MZ and AS, statistical analysis: MZ, writing—original draft preparation, MZ and AE; writing—review and editing, MZ and AE. All authors contributed to the article and approved the submitted version.
